# Recurrent renal hydatid disease with suspected pelvic seeding: a case report

**DOI:** 10.1093/jscr/rjaf735

**Published:** 2026-01-15

**Authors:** Ahmed Alanzi, Bano Alsaleh, Dawood Alatefi, Khaled M AlAani, Hosameldin Mohamad

**Affiliations:** Anaesthesia and Pain Management Department, King Hamad University Hospital, Building 2435, Road 2835, Block 228, PO Box 24343, Busaiteen, Kingdom of Bahrain; Radiology Department, King Hamad University Hospital, Building 2435, Road 2835, Block 228, PO Box 24343, Busaiteen, Kingdom of Bahrain; University of Jordan, Queen Rania Street, Amman 11942, Jordan; Arabian Gulf University, Building 293, Road 2904, Block 329, Manama 26671, Kingdom of Bahrain; Radiology Department, King Hamad University Hospital, Building 2435, Road 2835, Block 228, PO Box 24343, Busaiteen, Kingdom of Bahrain

**Keywords:** renal hydatid disease, echinococcosis, recurrent cystic echinococcosis, pelvic hydatid cyst, laparoscopic deroofing, multimodal imaging

## Abstract

Renal hydatid disease is a rare manifestation of echinococcosis, accounting for ˂4% of cases. We report a 47-year-old male with two prior surgeries for right renal hydatid cysts, presenting with right upper quadrant pain, dysuria, and low-grade fever. Imaging revealed a recurrent right renal cystic lesion and an incidental pelvic cyst near the seminal vesicle, raising concern for possible dissemination. Laparoscopic aspiration and deroofing of the renal cyst were performed, followed by a 6-month course of albendazole. Recovery was uneventful, and follow-up imaging showed no recurrence or new cyst formation. This case highlights the importance of long-term surveillance in renal hydatid disease and the need to consider rare sites of recurrence or dissemination. Multimodal imaging and minimally invasive surgery play a central role in diagnosis and management, even in patients outside endemic regions.

## Introduction

Echinococcosis is a parasitic zoonosis caused by *Echinococcus granulosus*, primarily endemic to the Middle East, Mediterranean region, South America, and parts of Asia and Africa [1]. Humans contract the infection via ingestion of parasitic eggs through contaminated food, water, or contact with infected dogs. The liver and lungs are the most commonly involved organs, but kidneys may also be affected in ⁓2%–4% of cases [2]. Renal hydatid cysts may remain asymptomatic for years due to their slow growth, and when symptomatic, may present with flank pain, hematuria, or hydatiduria. Diagnosis relies on imaging, particularly ultrasound and computed tomography (CT), supported by serology when available [3]. Treatment involves surgery with or without antiparasitic therapy, and recurrence can occur despite adequate management [4].

## Case presentation

A 47-year-old male presented with a 3-week history of right upper quadrant pain, nausea, vomiting, dysuria, low-grade fever, and reduced appetite. He denied weight loss, rectal bleeding, or recent travel to endemic areas. He had undergone laparoscopic deroofing of a right renal hydatid cyst in 2014 and again in 2022.

On examination, he was hemodynamically stable. Abdominal exam revealed right flank tenderness and a palpable mass. Laboratory investigations, including complete blood count, liver and renal function tests, and echinococcal serology, were within normal limits. Urinalysis and urine culture were negative.

**Figure 1:**
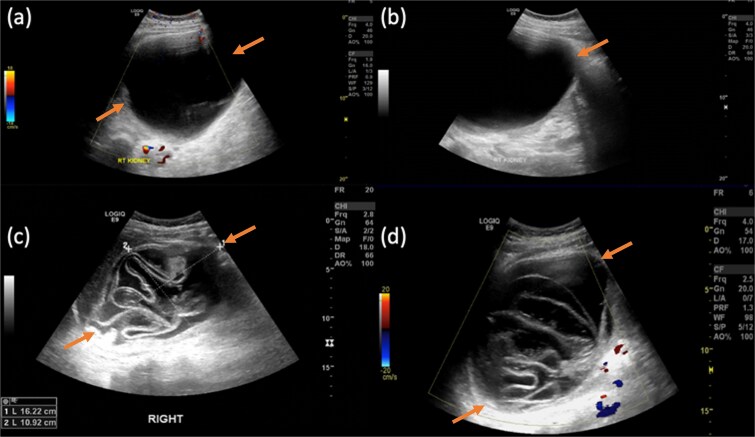
Gray-scale transabdominal ultrasound demonstrates a large well-defined anechoic lesion located in the upper pole of the kidney. The lesion contains internal, non-septated debris and shows no internal vascularity on color doppler imaging (a and b). Compared with previous right renal lesion showing collapsed membranes float within the cyst fluid, resembling water lily.

**Figure 2:**
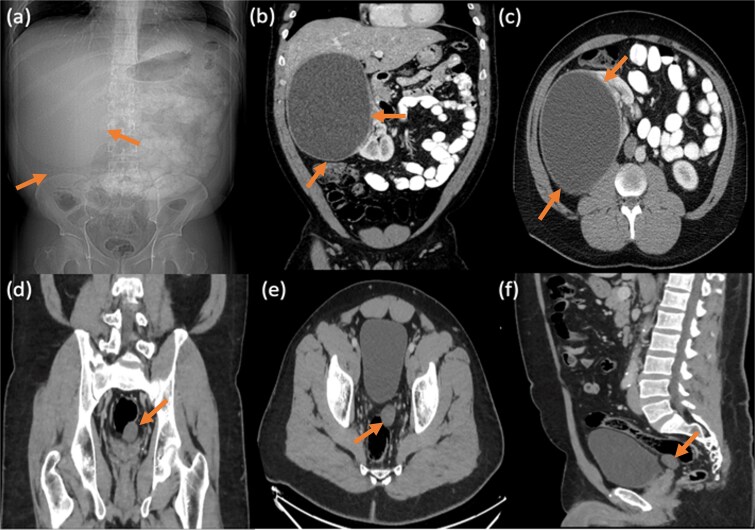
Frontal abdominal radiograph in supine position showing opacity occupying the right upper abdominal region with paucity of bowel loops (a). Followed by contrast enhanced CT abdomen and pelvis with reconstructed images showing a large thick walled non-septated non-calcific right renal cortical cyst (b and c). No right perinephric/pericystic fat stranding or free fluid, preserved tissue planes with surrounding related organs. Incidentally noted small similar looking cystic lesion in the left pelvic region, inseparable from the rectal wall and left seminal vesicle showing no gross fat or calcification (d–f).

Ultrasound showed a large cystic lesion in the right kidney with internal debris and fluid–fluid levels (Fig. 1). Contrast-enhanced CT confirmed a thick-walled, fluid-filled cyst in the right upper renal pole without septations or calcifications—consistent with recurrent hydatid disease (Fig. 2). Incidentally, a small pelvic cystic lesion was seen adjacent to the rectum and seminal vesicle.

**Figure 3:**
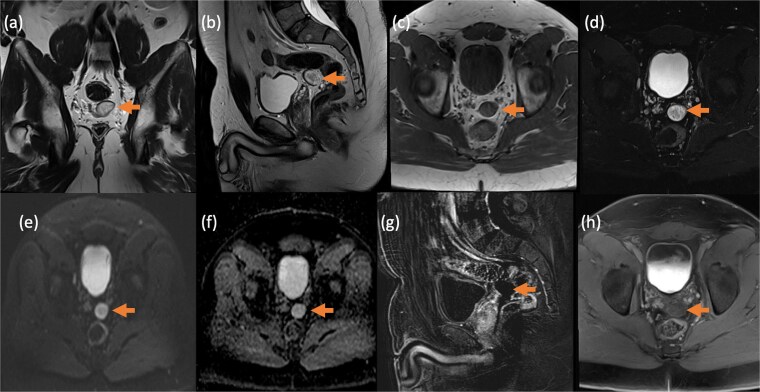
Contrast-enhanced MRI of the pelvis in different sequences and different planes demonstrates a small, well-defined, oval-shaped lesion with high signal intensity on T2-weighted images (bold arrow—a and b) and low signal intensity on T1-weighted images (bold arrow—c). The lesion is located in the pelvis, inseparable from the seminal vesicles and the anterior rectal wall. It shows no signal drop on fat-saturated T2 sequence (bold arrow—d) no signify macroscopic fat, no restricted diffusion (dashed arrow—e and f) with T2 shine through, and no post-contrast enhancement on early subtracted (arrow head—g) and no delayed enhancement (arrow head—h). No solid component.

**Figure 4:**
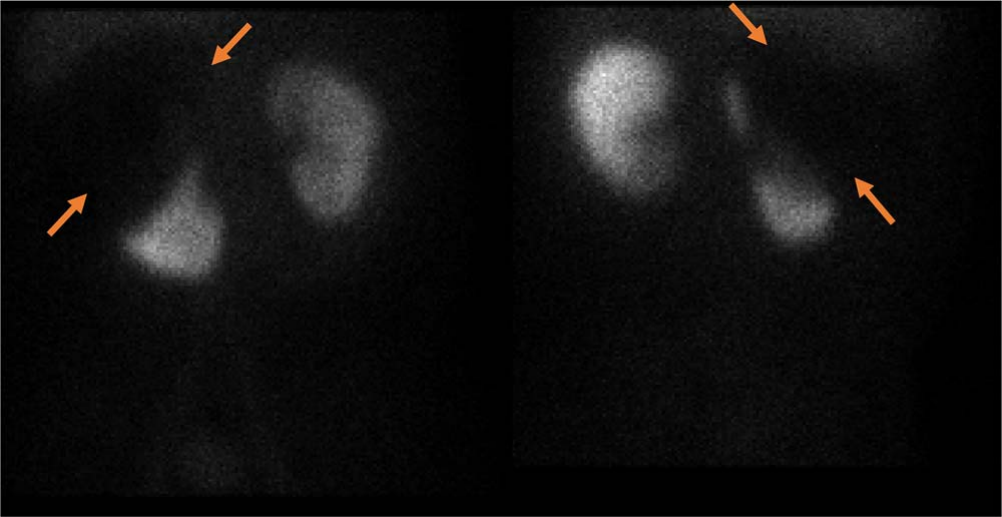
Static image of the abdomen at 2 h after IV injection TC-99 m DMSA, showing normal morphology & corticotubular function of the left kidney. Large photopenic area occupying the upper half of the right kidney while its lower half showed preserved cortical function.

Pelvic magnetic resonance imaging (MRI) further characterized the pelvic lesion as a well-defined, non-enhancing, fluid-filled cyst measuring 3 × 2 × 1 cm without solid components, raising suspicion for pelvic hydatid seeding, although peritoneal inclusion cyst was also considered (Fig. 3). A 99mTc-dimercaptosuccinic acid (DMSA) scan showed preserved renal function with the right kidney contributing 25.7% of total function and a photopenic defect at the cyst site (Fig. 4). No other lesions were found on chest CT or brain MRI. Colonoscopy ruled out malignancy but revealed incidental low-grade adenomas.

The patient underwent laparoscopic aspiration and deroofing of the renal cyst. Recovery was uneventful. He was started on albendazole 400 mg twice daily for 6 months with monthly liver function monitoring. At 3-month follow-up, he remained asymptomatic, and imaging showed no recurrence. The incidental pelvic cyst remained unchanged in size and appearance and, given its small size, lack of enhancement, and benign imaging features, it was managed conservatively with continued radiological surveillance.

## Discussion

Renal hydatid disease presents diagnostic challenges due to its rarity and often non-specific symptoms. In this case, serological tests were negative—a known limitation in isolated renal hydatid disease due to low sensitivity in localized infections [3]. Imaging played a pivotal role in diagnosis and surveillance. CT confirmed cystic recurrence, and MRI was essential in evaluating the pelvic lesion. Although rare, pelvic seeding may result from intraoperative spillage or rupture [2]. In our patient, the pelvic lesion was managed conservatively with serial imaging, as its stability and benign features supported observation rather than intervention.

MRI findings—high T2 signal, absence of enhancement, and lack of diffusion restriction—suggested a benign cyst. Yet, the patient’s surgical history warranted suspicion for secondary hydatid dissemination. This highlights MRI’s value in soft tissue evaluation where CT may be limited [5].

Functional imaging confirmed adequate residual function in the affected kidney, supporting a nephron-sparing surgical approach such as cyst excision or partial nephrectomy. Surgical management remains the cornerstone of treatment for renal hydatid disease, with laparoscopic techniques favored due to reduced morbidity and faster recovery [6, 7]. This minimally invasive approach is particularly advantageous in recurrent cases, where prior surgeries may result in adhesions and distorted anatomy.

Albendazole is the antiparasitic agent of choice. Preoperative therapy may sterilize the cyst, while postoperative treatment reduces recurrence risk by targeting residual or disseminated larvae [8, 9]. Our patient completed a 6-month course with regular liver function monitoring and no adverse effects.

Long-term follow-up is essential, as recurrence may occur years after surgery. This patient had two recurrences across nearly a decade, emphasizing the need for surveillance imaging and clinical monitoring. Although extra-renal dissemination is rare, reports have documented peritoneal, pelvic, and even central nervous system involvement [10].

## Conclusion

This case illustrates the recurrent and potentially disseminated nature of renal hydatid disease, the diagnostic limitations of serology, and the value of multimodal imaging. The incidental detection of a pelvic cyst underscores the importance of comprehensive evaluation. Laparoscopic management combined with albendazole therapy yielded a favorable outcome. Lifelong surveillance remains key to early detection of recurrence and prevention of complications.

## References

[1] Saadeddine Zmerli M, Mohsen Ayed M, Ali Horchani M, et al. Hydatid cyst of the kidney: diagnosis and treatment. World J Surg 2001;25:68–74. 10.1007/s002680020009 11213158

[2] Sachar S, Goyal S, Sangwan S. Uncommon locations and presentations of hydatid cyst. Ann Med Health Sci Res 2014;4:447–52. 10.4103/2141-9248.133476 24971224 PMC4071749

[3] Zait H, Hamrioui B. Human cystic echinococcosis: serological diagnosis by indirect hemagglutination test, enzyme-linked immunosorbent assay, immunoelectrophoresis, and immunoblotting in surgically confirmed patients versus cases diagnosed by imaging techniques. Med Mal Infect 2020;50:676–83. 10.1016/j.medmal.2019.10.001 31727467

[4] Rexiati M, Mutalifu A, Azhati B, et al. Diagnosis and surgical treatment of renal hydatid disease: a retrospective analysis of 30 cases. PloS One 2014;9:e96602. 10.1371/journal.pone.0096602 24796329 PMC4010549

[5] Shah A, Rojas CA. Imaging modalities (MRI, CT, PET/CT), indications, differential diagnosis and imaging characteristics of cystic mediastinal masses: a review. Mediastinum. 2022;7:3. 10.21037/med-22-31. 36926286 PMC10011868

[6] Leslie S, Goh AC, Gill IS. Partial nephrectomy—contemporary indications, techniques and outcomes. Nat Rev Urol 2013;10:275–83. 10.1038/nrurol.2013.69 23588406

[7] Kumar S, Singla S, Ahmad A, et al. Isolated renal hydatid disease: laparoscopic approach to an uncommon entity (case report). Pan African Med J 2021;38:175. 10.11604/pamj.2021.38.175.27993 PMC8077677 33995782

[8] Kappagoda S, Singh U, Blackburn BG, editors. Antiparasitic therapy. *Mayo Clin Proc* 2011;**86**:S1–112. 10.4065/mcp.2011.0203 PMC3104918 21628620

[9] Basnet P, Chapagain S, Neupane R, et al. Rare encounter of renal hydatid cyst: a case report. JNMA J Nepal Med Assoc 2021;59:716–18. 10.31729/jnma.6994 PMC9107858 34508491

[10] Velasco-Tirado V, Alonso-Sardón M, Lopez-Bernus A, et al. Medical treatment of cystic echinococcosis: systematic review and meta-analysis. BMC Infect Dis 2018;18:306. 10.1186/s12879-018-3201-y 29976137 PMC6034244

